# Healthy life expectancy and its trends among Brazilian older adults from 2003 to 2019: an observational study using PNAD and PNS data

**DOI:** 10.3389/fpubh.2026.1797715

**Published:** 2026-10-19

**Authors:** Vanovya Alves Claudino Cesário, Kenio Costa Lima

**Affiliations:** Health Sciences Center, Federal University of Rio Grande do Norte, Natal, Brazil

**Keywords:** functional capacity, health expectancy, healthy life expectancy, life expectancy, multimorbidity, self-rated health

## Abstract

**Introduction:**

Population longevity is a relatively recent phenomenon in Latin America. In Brazil, the region’s largest and most populous country, studies examining healthy life expectancy (HLE) are scarce. Hence, this repeated/serial cross-sectional study aimed to analyze healthy life expectancy (HLE) and its trends among Brazilian older adults at national and regional levels from 2003 to 2019. The analysis used data on functional disability, self-rated health, and multimorbidity from the Brazilian National Household Sample Survey (PNAD) and National Health Survey (PNS), 2013 and 2019, both conducted by the Brazilian Institute of Geography and Statistics (IBGE).

**Methods:**

The PNAD and PNS employed a three-stage cluster probability sampling design. The territorial divisions and sectoral networks established for the 2000 (PNAD 2003 and 2008) and 2010 (PNS 2013 and 2019) population censuses served as the primary and secondary sampling units, respectively. Health indicators were analyzed by estimating prevalence, stratified by sex, age group, and geographic region, with 95% confidence intervals, while accounting for the complex sampling design, sample weights, and design effects. Life expectancy (LE), disability-free life expectancy (DFLE), multimorbidity-free life expectancy (MFLE), and life expectancy with good self-rated health (GSR-LE) were calculated using Sullivan’s method and compared by sex for 2003 and 2019. The data used were secondary and publicly available; therefore, approval from an ethics committee was not required. The variables analyzed were sex, geographic region, self-rated health, functional disability, and multimorbidity.

**Results:**

Among Brazilian older adults, HLE (0.57%), DFLE (11.94%), MFLE (13.70%), and GSR-LE (18.91%) increased. Although women experienced a greater increase in LE, their gains in HLE were slightly lower than those of men across all analyzed dimensions, both nationally and across all geographic regions. Men showed an absolute compression of multimorbidity and a relative compression of LE with functional disability and poor self-rated health.

**Conclusion:**

These sex-based discrepancies reinforce the gender health survival paradox, highlighting the potential relationship between the findings and genetic, behavioral, and cultural factors, which could guide the formulation of public health policies.

## 1 Introduction

The World Health Organization (WHO) (1) reported a global increase of 2.3 years in life expectancy (LE) and 1.7 years in healthy life expectancy (HLE) at age 60 from 2000 to 2019. These indicators also increased across all South American countries during this period. Consistent with the global trend, Brazil’s older population experienced a 2.2-year increase in LE and a 1.6-year increase in HLE (2).

The WHO (3) defines HLE as the number of years a person is expected to live without disability (4), based on the understanding that the goal of healthy aging is to maintain functional capacity. However, the concept of “being healthy” requires a multidimensional analysis that considers the complexity of the term “health” and its trends. To this end, it is essential to analyze measures that address this complexity at the population level (5), such as the presence or absence of multimorbidity, (dis)ability regarding basic activities of daily living (BADL), and older adults’ self-rated health (6).

Analyzing additional years of life—specifically, multimorbidity-free life expectancy (MFLE), disability-free life expectancy (DFLE) regarding BADL, and life expectancy with good self-rated health (GSR-LE), both individually and in combination—allows for the identification of each dimension’s impact on the quality of life of older adults. It also informs the development of public policies designed to ensure their well-being (5).

Multimorbidity refers to a condition in which individuals present two or more chronic diseases simultaneously, including physical, infectious, or mental health conditions (7, 8). Self-rated health, whose relevance for health monitoring has been recognized worldwide (6), is a subjective measure reflecting how an individual perceives their overall health status. This perception encompasses the older adult’s physical, emotional, and mental capacities, their life expectations and daily experiences, and how they view themselves within society and their specific social group (9, 10). Functional capacity is linked to an individual’s independence (11), as it is assessed based on reported difficulties in performing BADL among older adults (12).

Analyzing multimorbidity, self-rated health, and functional capacity allows for the characterization of the health status of older adults. To this end, it is necessary to identify whether there has been an absolute compression (a decrease in the years of unhealthy life) or expansion (an increase in the years of unhealthy life) of the conditions under analysis (multimorbidity, functional disability, and poor self-rated health). Relative compression is characterized by a decrease in the proportion of unhealthy life (12, 13), while relative expansion is defined by an increase in the proportion of unhealthy life (12–14).

Given that longevity is a recent phenomenon in Latin America (15) and that few national studies address HLE in Brazil, further investigation is required. Brazil is the largest and most populous country in Latin America. Since 2003, it has expanded its commitment to addressing the needs of its older population by restructuring legal frameworks, policies, and programs specifically targeting this demographic group. Therefore, this study seeks to address a gap in the literature regarding the health conditions of Brazil’s older population in light of their increasing LE.

Thus, this study aimed to analyze HLE and its trends among Brazilian older adults at national and regional levels from 2003 to 2019. Using HLE (a summary measure incorporating morbidity and mortality) (16) allows for a deeper understanding of the aging process of the Brazilian population and the quality of the additional years lived by older adults, thereby fostering the development of initiatives aimed at improving the quality of life of older adults and their families.

## 2 Materials and methods

### 2.1 Study design

This was a repeated/serial (17) population-based cross-sectional study using secondary data from independent cross-sectional studies that are publicly available and do not require ethics committee approval. It analyzed a historical HLE series at age 60 from 2003 to 2019. The unit of analysis comprised all Brazilian older adults who participated in the Brazilian National Household Sample Survey (PNAD) in 2003 (35,110 older adults) and 2008 (41,269 older adults) and in the Brazilian National Health Survey (PNS) in 2013 (11,177 older adults) and 2019 (22,728 older adults). These population-based surveys are representative of Brazil and its regions (North, Northeast, Central-West, South, and Southeast) and were conducted by the Brazilian Institute of Geography and Statistics (IBGE) in partnership with the Ministry of Health (10, 18, 19). Individual microdata from these surveys are available on the official IBGE website (20, 21). The IBGE is Brazil’s official national agency responsible for producing geographic, statistical, and environmental data on the country and its population (22).

### 2.2 Data source

The PNAD and PNS are conducted every 5 years. PNAD data on the health characteristics and physical mobility of residents aged 14 years and older are derived from the Health Supplement questionnaire (see Supplementary Figure 1). PNS data are derived from the questionnaire’s Module K (health of individuals aged 60 years and older and mammography coverage among women aged 50 years and older), Module N (perception of health status), and Module Q (chronic diseases).

The first edition of the PNS, specifically focused on health, was conducted in 2013 to refine and expand upon studies based on data from the PNAD Health Supplement (10, 19). Certain factors were maintained, such as the survey frequency, the monitoring of previously analyzed indicators, and the use of the PNAD master sample, given that this survey is part of the IBGE’s Integrated System of Household Surveys (10, 19).

These surveys employed a three-stage probability cluster sampling design. In the first stage, the IBGE master sample was used to define the primary sampling unit. In the second stage, municipalities or census tracts with probabilities proportional to the current population were selected, with replacement in the PNAD and through simple random sampling in the PNS. In the third stage, households were selected for the survey with equal probability in the PNAD and through simple random sampling from the list of household residents in the PNS. The selection of the primary and secondary units was based on the territorial divisions and census tract networks established for the 2000 (PNAD 2003 and 2008) and 2010 (PNS 2013 and 2019) population censuses (10, 18).

Comparability between the PNAD and the PNS was ensured by maintaining the fundamental aspects of the surveys, thereby enabling the continuous updating of population health data, contributing to the formulation, monitoring, and evaluation of such policies, and supporting the development of public health research (10, 17).

To this end, as shown in Supplementary Figure 2, and to minimize residual non-comparability bias (characterized by the underestimation or overestimation of results), the study performed ex post harmonization of data from the PNAD Health Supplement (2003 and 2008) and the PNS (2013 and 2019) across the study period.

Self-rated health was divided into “good” (summing “very good” and “good” responses) and “poor” (summing “fair,” “poor,” and “very poor” responses). This measure demonstrated high comparability, as the categories and question wording remained unchanged, thereby minimizing residual bias. Multimorbidity, defined as the presence of two or more conditions in older adults, was assessed by selecting conditions common to all four surveys, based on a study by a research group in Pelotas that established an official list of chronic diseases for analyzing multimorbidity in Brazil (22). However, residual positive bias persists due to improved diagnostic identification in the PNS.

The study also harmonized functional disability to ensure comparability by including only identical or corresponding BADL items across all surveys. Disability status was divided into “no disability” (for individuals reporting “no difficulty” with any BADL) and “functional disability” (for individuals reporting inability, great difficulty, or some difficulty with at least one BADL). However, the PNS tends to yield higher prevalence rates of functional disability than the PNAD.

The harmonization process applied to the health dimensions may have influenced the magnitude of the observed trends. Analyzing multimorbidity by focusing on the common chronic diseases included in all four surveys (rather than the broader set of conditions available in the 2013 and 2019 datasets) may have yielded conservative estimates, with prevalence rates lower than those that would have been identified had a wider spectrum of chronic conditions been included. However, including conditions available only in the PNS could have increased the prevalence of multimorbidity and either altered or attenuated the estimated MFLE magnitude. Furthermore, differences in the questions used to assess functional disability and self-rated health across the PNAD and PNS may have introduced some residual measurement discrepancies. Thus, while the possibility of uncertainty regarding the observed changes must be acknowledged, it is essential to emphasize that the harmonization strategies employed were primarily designed to maximize comparability across survey waves and minimize potential measurement differences.

### 2.3 Data collection

Health dimensions were identified based on responses to questions from the PNAD Health Supplement in 2003 and 2008 and from the PNS, a dedicated health survey conducted in 2013 and, as a government initiative, in 2019 (with data released in 2020 and 2021).

Computer-assisted personal interviews were conducted in person at the respondents’ homes by teams previously trained by the IBGE. The selected questions (described in Supplementary Figure 1) addressed older adults’ self-rated health status, functional capacity (analyzed based on responses concerning difficulties with eating, bathing, and using the toilet due to health issues), and multimorbidity (assessed using questions on diagnoses or professional reports regarding the presence of specific selected diseases).

The absence of an official list of chronic diseases for multimorbidity analysis in Brazil prompted a group of researchers from the Federal University of Pelotas to develop one. This list was used to identify diseases that appeared in both the PNAD and PNS and were included in this study, such as rheumatoid arthritis, cancer, diabetes mellitus, asthmatic bronchitis, systemic arterial hypertension, heart disease, kidney disease or chronic renal failure, and depression (23).

### 2.4 Health dimensions

Good self-rated health was identified as a self-rating of “very good” or “good,” while ratings of “fair,” “poor,” or “very poor” were categorized as poor self-rated health. Functional capacity was analyzed based on BADL. Older adults who reported any degree of difficulty (unable to perform, great difficulty, or slight difficulty) with at least one BADL were considered to have functional disability, whereas those who reported “no difficulty” with any BADL were considered free of functional disability. Older adults categorized as having multimorbidity were those who answered “yes” when asked if they had or had been diagnosed with two or more selected chronic diseases.

### 2.5 Statistical analysis

The time series data for Brazilian older adults were analyzed by sex, health dimension, and geographic region and at the national level, with prevalence estimates and 95% confidence intervals. The analysis was performed using the “survey” package in R and accounted for the complex sampling design, sample weights, and design effects to ensure the validity of the estimates and their representativeness for the Brazilian population. This approach allows for the inference that the statistical estimates are generalizable to the target population.

HLE was estimated using Sullivan’s method (24, 25) by combining IBGE complete life tables for 2003, 2008, 2013, and 2019 (disaggregated by region and sex where applicable), population mortality data for that period, and the prevalence of each health dimension considered (24, 25). The latter is suitable for the present study due to its relative precision and ease of interpretation; furthermore, it relies on cross-sectional data, enabling comparisons of the health status of an entire population at two different time points or of two different populations at the same time point (24). However, it is not a predictive model, as it reflects only the health status observed at the time of measurement.

HLE was calculated at the national level using national complete life tables and by geographic region using regional life tables for the respective years. These dimensions were aggregated into a single measure (HLE), considering older adults who were simultaneously free from multimorbidity and BADL disability and who reported good self-rated health. The same approach was used to calculate DFLE, MFLE, and GSR-LE.

Sullivan’s method estimates HLE by subtracting the years lived in poor health from the total LE of older adults, using the following formula:

$$\mathrm{HL}E_{X}=\frac{\sum (n\pi x)nL_{x}}{l_{x}}$$

HLE, calculated using individual-level microdata, is the average number of years lived in good health (free from functional disability, free from multimorbidity, and/or with good self-rated health), starting from age x. In the formula, n represents the length of the age interval (5 years), with an open-ended interval at age 80; _n_𝝅_x_ is the prevalence of good health (free from functional disability, free from multimorbidity, and/or with good self-rated health) in the age group x to x + n; _n_L_x_ is the total number of person-years lived by the cohort during the interval; and l_x_ is the probability of surviving to age x (24, 26).

The 95% confidence intervals for health expectations were calculated using the prevalence estimates of HLE, DFLE, MFLE, and GSR-LE. Then, the study compared healthy life expectancies in 2003 and 2019 nationally and regionally, by sex, based on life expectancies and their variances (variance formula), to verify inequalities in HLE, DFLE, MFLE, and GSR-LE, based on the Sullivan method calculation as described by Jagger et al. (24), using the multinomial variance formula.

The study also estimated the proportion of healthy life, as well as the number of healthy and unhealthy years, over the period from 2003 (marking the expansion of legal frameworks, programs, and policies targeting the Brazilian older population) to 2019, the year for which the most recent official data on the health of the Brazilian population were available. The observed differences made it possible to assess the health status of the older population throughout the analyzed period and to identify potential patterns of HLE compression or expansion among Brazilian individuals over the years.

## 3 Results

### 3.1 Sociodemographic characterization

Throughout the analyzed period, the socioeconomic and demographic characteristics of the population under study remained stable, with a predominance of White, female, literate individuals aged 60 to 69 years and living in Southeastern Brazil. Notably, there was a 21% increase in the percentage of older adults who reported being literate in 2019 compared to 2003 (81.4% vs. 67.4%), as shown in Table 1.

**Table 1:** Distribution of the Brazilian older population according to sociodemographic characteristics and health dimensions by year.

Sociodemographic characteristics and health dimensions	2003% (95% CI) *n* = 35,110	2008% (95% CI) *n* = 41,269	2013% (95% CI) *n* = 11,177	2019% (95% CI) *n* = 22,728
Sex				
Male	44.1 (43.6 to 44.5)	43.8 (43.4 to 44.2)	43.6 (42.1 to 45.2)	43.3 (42.3 to 44.4)
Female	55.9 (55.5 to 56.4)	56.2 (55.8 to 56.6)	56.4 (54.8 to 57.9)	56.7 (55.6 to 57.7)
Race				
Indigenous Brazilian	0.2 (0.1 to 0.3)	0.3 (0.2 to 0.4)	0.3 (0.2 to 0.4)	0.5 (0.4 to 0.7)
White	59.1 (58.3 to 59.9)	56.1 (55.3 to 56.8)	53.8 (52.1 to 55.4)	50.5 (49.4 to 51.7)
Black	6.8 (6.4 to 7.1)	7.4 (7 to 7.7)	9.2 (8.3 to 10.2)	10.3 (9.6 to 11)
East Asian	0.9 (0.7 to 1.1)	1.1 (1 to 1.3)	1.2 (0.9 to 1.6)	1.3 (1 to 1.6)
Mixed Race	33 (32.3 to 33.8)	35.1 (34.3 to 35.8)	35.6 (34 to 37.1)	37.4 (36.3 to 38.5)
Geographic region				
North	3.7 (3.5 to 3.9)	5.2 (4.9 to 5.6)	5.4 (5 to 5.9)	6.1 (5.8 to 6.4)
Northeast	27.4 (26.7 to 28.1)	25.6 (25.1 to 26.2)	25.1 (24 to 26.2)	25.4 (24.6 to 26.2)
Southeast	47.3 (46.6 to 48.1)	47.4 (46.7 to 48.1)	48 (46.6 to 49.5)	46.4 (45.4 to 47.5)
South	16 (15.5 to 16.6)	15.7 (15.2 to 16.3)	15 (14.2 to 16)	15.7 (15.1 to 16.4)
Central-West	5.5 (5.3 to 5.8)	6 (5.7 to 6.2)	6.4 (6 to 6.9)	6.4 (6 to 6.7)
Age group (years)				
60 to 64	31.1 (30.5 to 31.6)	30.9 (30.4 to 31.5)	32 (30.5 to 33.5)	31.1 (30.1 to 32.1)
65 to 69	24.4 (23.9 to 24.9)	24.5 (24.1 to 25)	24.6 (23.3 to 25.9)	25.2 (24.3 to 26.2)
70 to 74	19.3 (18.9 to 19.8)	18.3 (17.9 to 18.7)	18.3 (17.1 to 19.5)	18.1 (17.3 to 18.9)
75 to 79	12.7 (12.3 to 13.1)	12.8 (12.4 to 13.2)	11.6 (10.7 to 12.6)	12 (11.4 to 12.8)
80 or older	12.5 (12 to 12.9)	13.4 (13.1 to 13.8)	13.6 (12.6 to 14.7)	13.6 (12.8 to 14.3)
Literate				
Yes	67.4 (66.6 to 68.2)	72 (71.3 to 72.7)	77.5 (76.2 to 78.7)	81.4 (80.5 to 82.2)
No	32.6 (31.8 to 33.4)	28 (27.3 to 28.7)	22.5 (21.3 to 23.8)	18.6 (17.8 to 19.5)
Poor self-rated health				
Yes	56.4 (55.6 to 57.2)	55 (54.3 to 55.7)	55.6 (54.1 to 57.1)	52.9 (51.8 to 54.1)
No	43.6 (42.8 to 44.4)	45 (44.3 to 45.7)	44.4 (42.9 to 45.9)	47.1 (45.9 to 48.2)
Functional disability				
Yes	13.5 (12.9 to 14)	15.2 (14.7 to 15.7)	9.4 (8.5 to 10.3)	12 (11.3 to 12.7)
No	86.5 (86 to 87.1)	84.8 (84.3 to 85.3)	90.6 (89.7 to 91.5)	88 (87.3 to 88.7)
Multimorbidity				
Yes	37 (36.3 to 37.8)	37.7 (37.1 to 38.3)	34.4 (32.8 to 36)	34.9 (33.8 to 36)
No	63 (62.2 to 63.7)	62.3 (61.7 to 62.9)	65.6 (64 to 67.2)	65.1 (64 to 66.2)

Source: The National Household Sample Survey (2003, 2008) and the National Health Survey (2013, 2019).

The North region showed a 64.86% increase, while the Central-West region showed a 16.36% increase in the representation of the older adult population. The proportions of individuals self-identifying as Black or mixed-race increased by 51.47 and 13.33%, respectively. The prevalence of functional disability, poor self-rated health, and multimorbidity in the Brazilian population decreased (Table 1).

### 3.2 Healthy life expectancy in Brazil and the major regions

The health status of the population analyzed differed between the Southeast and other Brazilian regions; the former consistently showed higher DFLE and HLE throughout the study period. The South and Southeast regions exhibited higher GSR-LE than the North and Northeast regions throughout the period analyzed. It is worth noting that the South region recorded the largest percentage increase in GSR-LE (30.46%), DFLE (18.69%), and HLE (29.15%). The North region recorded the greatest increase (35.75%) in MFLE and the second-largest increase in HLE (23.73%) between 2003 and 2019 (Table 2).

**Table 2:** Healthy life expectancy according to self-rated health, functional disability, and multimorbidity among the Brazilian older population from 2003 to 2019.

Healthy life expectancy by health dimension	2003 years (95% CI)	2008 years (95% CI)	2013 years (95% CI)	2019 years (95% CI)
Brazil				
GSR-LE	8.99 (8.83 to 9.16)	9.52 (9.37 to 9.67)	9.68 (9.35 to 10)	10.69 (10.41 to 10.94)
DFLE	17.84(17.74 to 17.96)	17.94 (17.84 to 18.05)	19.75(19.55 to 19.94)	19.97 (19.81 to 20.13)
MFLE	12.99 (12.83 to 13.14)	13.18 (13.05 to 13.31)	14.30 (13.95 to 14.65)	14.77 (14.52 to 15.02)
HLE	7.03 (6.87 to 7.20)	7.24 (7.11 to 7.36)	7.37 (7.06 to 7.69)	7.78 (7.53 to 8.03)
North				
GSR-LE	6.79 (6.26 to 7.36)	7.28 (6.76 to 7.83)	7.12 (6.28 to 7.98)	8.24 (7.66 to 8.82)
DFLE	15.78 (15.21 to 16.26)	16.47 (16.16 to 16.74)	17.70 (16.95 to 18.30)	18.15 (17.74 to 18.51)
MFLE	11.19 (10.66 to 11.71)	12.48 (12.11 to 12.84)	15.29 (14.46 to 16.05)	15.19 (14.71 to 15.64)
HLE	5.14 (4.66 to 5.67)	5.80 (5.39 to 6.23)	5.74 (4.97 to 6.58)	6.36 (5.88 to 6.87)
Northeast				
GSR-LE	6.70 (6.36 to 7.03)	7.38 (7.18 to 7.60)	7.21 (6.71 to 7.71)	7.54 (7.15 to 7.93)
DFLE	16.24(16.01 to 16.47)	16.44 (16.23 to 16.66)	18.60 (18.31 to 18.87)	18.60 (18.34 to 18.84)
MFLE	13.02 (12.72 to 13.33)	13.57 (13.35 to 13.79)	15.06 (14.54 to 15.56)	14.74 (14.37 to 15.08)
HLE	5.51 (5.19 to 5.84)	5.81 (5.61 to 5.99)	5.74 (5.26 to 6.23)	5.93 (5.56 to 6.29)
Southeast				
GSR-LE	9.92 (9.69 to 10.16)	10.74 (10.50 to 10.95)	11.24 (10.66 to 11.84)	12.46 (11.97 to 12.97)
DFLE	17.86 (17.71 to 18.02)	18.46(18.31 to 18.61)	20.51 (20.15 to 20.82)	20.69 (20.41 to 20.97)
MFLE	12.73 (12.52 to 12.91)	13.05(12.84 to 13.25)	14.01 (13.41 to 14.60)	14.79 (14.32 to 15.23)
HLE	7.78 (7.57 to 8)	8.10 (7.91 to 8.32)	8.44 (7.93 to 9)	8.72 (8.28 to 9.19)
South				
GSR-LE	9.06 (8.62 to 9.48)	9.86 (9.48 to 10.27)	10.21 (9.45 to 10.99)	11.82 (11.26 to 12.41)
DFLE	17.34 (17.12 to 17.54)	18.02(17.74 to 18.25)	20.19 (19.66 to 20.62)	20.58 (20.21 to 20.93)
MFLE	11.41 (11.07 to 11.77)	12.18 (11.86 to 12.48)	13.36 (12.56 to 14.15)	14.45 (13.91 to 14.99)
HLE	6.62 (6.26 to 7)	7.22 (6.90 to 7.54)	7.25 (6.51 to 8.01)	8.55 (7.98 to 9.13)
Central-West				
GSR-LE	8.16 (7.73 to 8.62)	8.69 (8.30 to 9.10)	9.24 (8.54 to 9.99)	10.26 (9.60 to 10.90)
DFLE	16.72 (16.28 to 17.10)	17.50 (17.19 to 17.79)	19.10 (18.52 to 19.57)	19.74 (19.35 to 20.10)
MFLE	11.87 (11.45 to 12.28)	12.49 (12.10 to 12.86)	13.98 (13.21 to 14.70)	14.14 (13.54 to 14.71)
HLE	6.24 (5.84 to 6.65)	6.52 (6.17 to 6.87)	7.60 (6.87 to 8.32)	7.50 (6.93 to 8.10)

*GSR-LE good, life expectancy with good self-rated health; DFLE, disability-free life expectancy for basic activities of daily living; MFLE, multimorbidity-free life expectancy; HLE, healthy life expectancy.

Source: The National Household Sample Survey (2003, 2008) and the National Health Survey (2013, 2019).

The increase in LE across all age groups between 2003 and 2019 was accompanied by a 10.67% increase in aggregate HLE and in all health dimensions analyzed (↑11.94% in DFLE, ↑18.91% in GSR-LE, and ↑13.70% in MFLE), as seen in Table 2. A more pronounced increase in unhealthy life expectancy (ULE) was identified in the population aged over 75 years from 2003 to 2019 (Figure 1; Supplementary Table 1).

**Figure 1:**
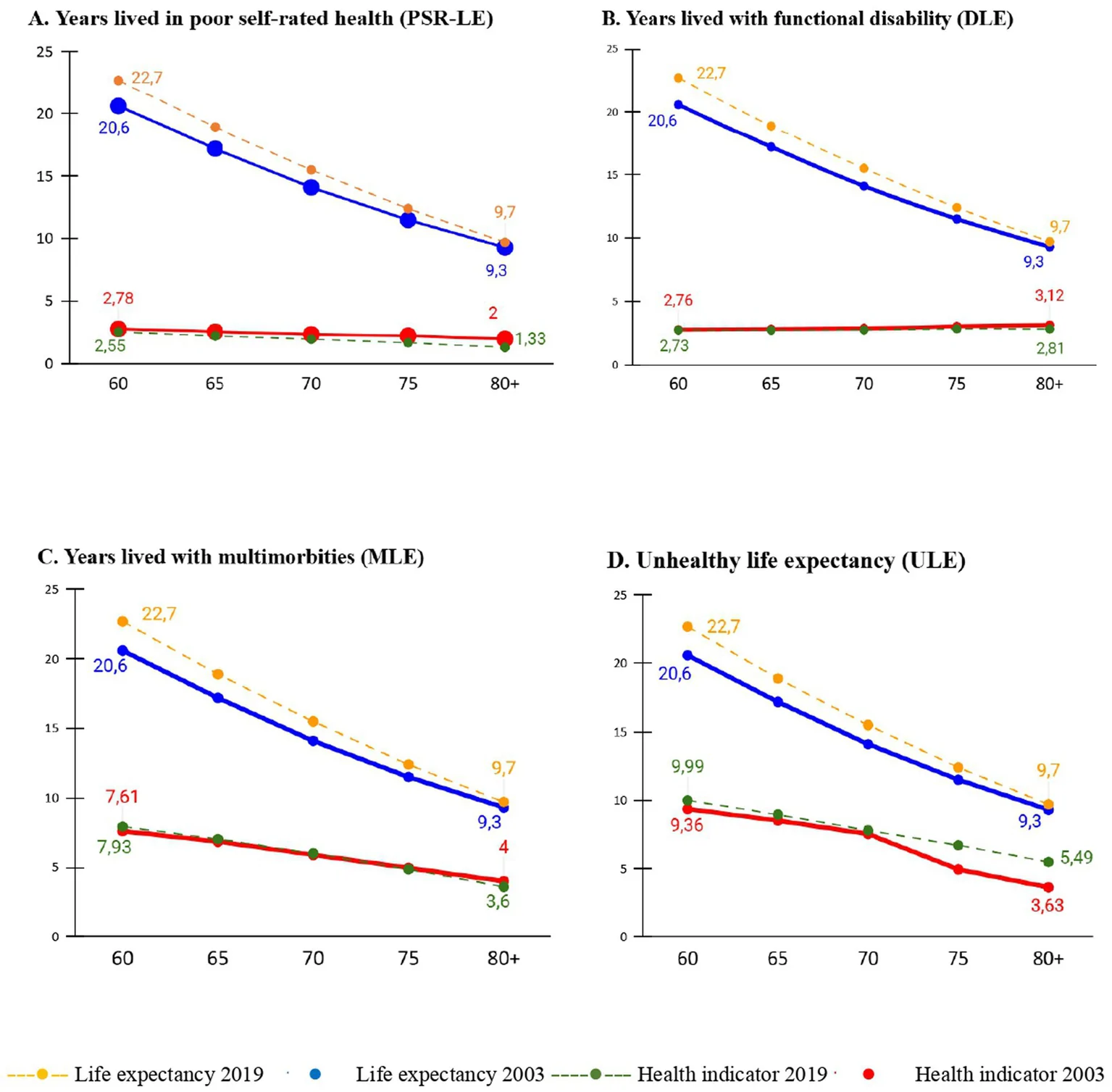
Life expectancy and years lived in selected health states in Brazil, 2003 and 2019. The figure distinguishes life expectancy in 2003 and 2019 and the health indicator in 2003 and 2019, as indicated by the corresponding symbols and line styles. Sources: The National Household Sample Survey (PNAD) 2003 and the National Health Survey (PNS) 2019.

A comparison between 2019 and 2003 revealed a relative compression of functional disability, driven by a 1.36% increase [95% CI: 0.70 to 2.02] in the proportion of years lived without functional disability. Conversely, there was an absolute expansion in ULE (↑1.35 years [95% CI: 1.15 to 1.55] in ULE), multimorbidity (↑0.32 years [95% CI: 0.12 to 0.53] in life expectancy with multimorbidity [MLE]), and poor self-rated health (↑0.40 years [95% CI: 0.17 to 0.64]) in life expectancy with poor self-rated health (PSR-LE), as shown in Table 3.

**Table 3:** Healthy life expectancy (HLE), disability-free life expectancy (DFLE), multimorbidity-free life expectancy (MFLE), and life expectancy with good self-rated health (GSR-LE) among the Brazilian older population from 2003 to 2019.

Health dimensions	2003	2019	Difference between 2019 and 2003
Healthy life expectancy			
Life expectancy (years)^1^	20.6	22.7	2.1
HLE (95% CI)^2^	7.03 (6.87 to 7.20)	7.78 (7.53 to 8.03)	0.75 (0.15 to 1.35)
Proportion of healthy life [(HLE/LE)*100]	34.14 (33.34 to 34.94)	34.28 (33.18 to 35.38)	0.15 (−0.06 to 0.35)
Unhealthy years^(1–2)^	13.57 (13.40 to 13.73)	14.92 (14.67 to 15.17)	1.35 (1.15 to 1.55)
Functional disability			
DFLE (95% CI)^3^	17.84 (17.74 to 17.96)	19.97 (19.81 to 20.13)	2.13 (1.98 to 2.27)
Proportion of life without disability	86.60 (86.10 to 87.20)	87.96 (87.26 to 88.66)	1.36 (0.70 to 2.02)
Years with disability^(1–3)^	2.76 (2.64 to 2.86)	2.73 (2.57 to 2.89)	−0.03 (−0.17 to 0.12)
Multimorbidity			
MFLE (95% CI)^4^	12.99 (12.83 to 13.14)	14.77 (14.52 to 15.02)	1.78 (1.57 to 1.98)
Proportion of life without multimorbidity	63.07 (62.27 to 63.77)	65.07 (63.97 to 66.17)	2 (1.05 to 2.95)
Years with multimorbidity^(1–4)^	7.61 (7.46 to 7.77)	7.93 (7.68 to 8.18)	0.32 (0.12 to 0.53)
Self-rated health			
GSR-LE (95% CI)^5^	8.99 (8.83 to 9.16)	10.69 (10.41 to 10.94)	1.69 (1.46 to 1.93)
Proportion of life free from poor health conditions	43.65 (42.85 to 44.45)	47.08 (45.88 to 48.18)	3.43 (3.19 to 3.67)
Years in poor health^(1–5)^	11.61 (11.44 to 11.77)	12.01 (11.76 to 12.29)	0.40 (0.17 to 0.64)

*Years with disability (DLE) = Life expectancy - DFLE^(1–3)^; Years with multimorbidity (MLE) = Life expectancy - MFLE^(1–4)^; Years in poor health (PSR-LE) = Life expectancy - GSR-LE^(1–5)^. ** Proportion of healthy life = (HLE/LE)*100.

Source: The National Household Sample Survey (2003) and the National Health Survey (2019).

### 3.3 Healthy life expectancy by sex

Women showed the largest statistically significant increases in unhealthy years across all regions compared to men, most notably in the Northeast region (↑2.17 years [95% CI 1.74–2.60]). Men showed a statistically significant absolute expansion in unhealthy years in the Northeast (1.50 years [95% CI 1.05–1.95]), Southeast (1.83 years [95% CI 1.27–2.39]), and South (1.45 years [95% CI 0.72–2.17]) regions.

Moreover, women experienced a greater increase in LE and HLE than men during the period analyzed. Table 4 shows that women experienced a statistically significant increase in HLE throughout this period, although these additional years also correspond to a greater increase in unhealthy life years, both nationally and across all Brazilian regions.

**Table 4:** Healthy life expectancy (HLE) according to self-rated health, functional disability, and multimorbidity among the Brazilian older population by sex and geographic region from 2003 to 2019.

Geographic region	2003		2019		Difference between 2019 and 2003	
	Female	Male	Female	Male	Female	Male
Brazil						
Life expectancy (years)^1^	22.1	19.1	24.4	20.7	2.3	1.6
HLE (95% CI)^2^	6.66 (6.49 to 6.86)	7.43 (7.24 to 7 0.62)	7.43 (7.0 8 to 7.77)	8.17 (7.83 to 8.48)	0.76 (0.48 to 1.05)	0.73 (0.44 to 1.02)
Healthy life proportion	30.2 (29.4 to 31.1)	38.91 (37.92 to 39.91)	30.44 (29.04 to 31.84)	39.45 (37.85 to 40.95)	0.29 (0.01 to 0.57)	0.54 (0.25 to 0.83)
Unhealthy years (ULE)^(1–2)^	15.44 (15.24 to 15.61)	11.67 (11.48 to 11.86)	16.97 (16.63 to 17.31)	12.53 (12.22 to 12.86)	1.53 (1.25 to 1.82)	0.87 (0.58 to 1.16)
North						
Life expectancy (years)^3^	19.64	17.19	22.41	19.17	2.77	1.98
HLE (95% CI)^4^	5.07 (4.52 to 5.64)	5.26 (4.61 to 5.96)	6.61 (5.85 to 7.40)	7.03 (6.32 to 7.78)	1.54 (0.77 to 2.32)	1.77 (1.03 to 2.52)
Healthy life proportion	25.81 (23.01 to 28.71)	30.60 (26.8 to 34.7)	29.51 (26.11 to 33.01)	36.70 (33 to 40.60)	3.70 (2.93 to 4.47)	6.09 (5.33 to 6.84)
Unhealthy years (ULE) ^(3–4)^	14.57 (14 to 15.12)	11.93 (11.22 to 12.58)	15.80 (15.01 to 16.56)	12.13 (11.39 to 12.84)	1.23 (0.45 to 2)	0.21 (−0.54 to 0.95)
Northeast						
Life expectancy (years)^5^	20.68	17.54	23.26	19.45	2.58	1.91
HLE (95% CI)^6^	5.15 (4.82 to 5.52)	5.87 (5.51 to 6.24)	5.56 (5.07 to 6.09)	6.28 (5.82 to 6.75)	0.41 (−0.02 to 0.84)	0.41 (−0.04 to 0.86)
Healthy life proportion	24.90 (23.30 to 26.70)	33.49 (31.39 to 35.59)	23.90 (21.8 to 26.2)	32.30 (29.9 to 34.7)	−0.99 (−1.43 to −0.56)	−1.19 (−1.65 to −0.74)
Unhealthy years (ULE)^(5–6)^	15.53 (15.16 to 15.86)	11.66 (11.30 to 12.03)	17.70 (17.16 to 18.19)	13.17 (12.7 to 13.63)	2.17 (1.74 to 2.60)	1.50 (1.05 to 1.95)
Southeast						
Life expectancy (years)^7^	22.18	18.32	24.98	21.39	2.8	3.07
HLE (95% CI)^8^	7.54 (7.27 to 7.83)	8.02 (7.77 to 8.30)	8.27 (7.67 to 8.89)	9.26 (8.66 to 9.86)	0.73 (0.19 to 1.27)	1.24 (0.68 to 1.80)
Healthy life proportion	33.99 (32.79 to 35.29)	43.79 (42.40 to 45.29)	33.10 (30.70 to 35.6)	43.3 (40.51 to 46.11)	−0.89 (−1.43 to −0.35)	−0.49 (−0.88 to −0.18)
Unhealthy years (ULE)^(7–8)^	14.64 (14.35 to 14.91)	10.30 (10.02 to 10.55)	16.71 (16.09 to 17.31)	12.13 (11.53 to 12.73)	2.07 (1.53 to 2.61)	1.83 (1.27 to 2.39)
South						
Life expectancy (years)^9^	21.71	18.03	25.33	21.29	3.62	3.26
HLE (95% CI)^10^	6.40 (5.93 to 6.88)	6.83 (6.44 to 7.27)	8.41 (7.57 to 9.27)	8.66 (7.96 to 9.37)	2 (1.27 to 2.74)	1.81 (1.09 to 2.54)
Healthy life proportion	29.50 (27.30 to 31.70)	38.01 (35.71 to 40.31)	33.19 (29.89 to 36.59)	40.70 (37.14 to 44)	3.70 (2.97 to 4.43)	2.69 (1.96 to 3.42)
Unhealthy years (ULE)^(9–10)^	15.31 (14.83 to 15.78)	11.18 (10.76 to 11.59)	16.93 (16.06 to 17.76)	12.63 (11.92 to 13.33)	1.62 (0.88 to 2.35)	1.45 (0.72 to 2.17)
Central-West						
Life expectancy (years)^11^	21.2	18.5	23.68	20.39	2.48	1.89
HLE (95% CI)^12^	5.87 (5.43 to 6.32)	6.59 (6.05 to 7.16)	7.06 (6.18 to 7.98)	7.99 (7.19 to 8.81)	1.18 (0.30 to 2.07)	1.41 (0.52 to 2.29)
Healthy life proportion	27.70 (25.60 to 29.80)	35.60 (32.7 to 38.7)	29.80 (26.10 to 33.7)	39.20 (35.3 to 43.2)	2.10 (1.21 to 2.98)	3.60 (2.70 to 4.50)
Unhealthy years (ULE)^(11–12)^	15.33 (14.88 to 15.77)	11.91 (11.34 to 12.45)	16.62 (15.70 to 17.5)	12.40 (11.58 to 13.19)	1.30 (0.41 to 2.18)	0.48 (−0.40 to 0.37)

*Superscript numbers identify the corresponding estimates used to calculate healthy life expectancy (HLE) and unhealthy life expectancy (ULE). HLE estimates are presented with 95% confidence intervals (CIs). ULE was calculated as the difference between life expectancy and HLE.

**Healthy life proportion = (HLE/LE)*100. Source: The National Household Sample Survey (2003) and the National Health Survey (2019).

In addition, the differences in HLE (except in the Northeast region), GSR-LE, DFLE, and MFLE between men and women were statistically significant during the period in question (Tables 4, 5).

**Table 5:** Disability-free life expectancy (DFLE), multimorbidity-free life expectancy (MFLE), and life expectancy with good self-rated health (GSR-LE) among the Brazilian older population by sex and geographic region from 2003 to 2019.

Health dimensions	2003		2019		Difference between 2019 and 2003	
	Female	Male	Female	Male	Female	Male
Functional disability						
Life expectancy (years)^1^	22.1	19.1	24.4	20.7	2.3	1.6
DFLE (95% CI)^2^	18.75 (18.60 to 18.90)	16.87 (16.75 to 16.98)	21.16 (20.91 to 21.40)	18.63 (18.43 to 18.82)	2.40 (2.19 to 2.62)	1.77 (1.58 to 1.95)
Proportion of life without disability	84.85 (84.15 to 85.54)	88.30 (87.7 to 88.9)	86.71 (85.7 to 87.7)	90.02 (89.01 to 90.92)	1.86 (0.93 to 2.79)	1.71 (0.78 to 2.64)
Years with disability^(1-2)^	3.35 (3.20 to 3.50)	2.23 (2.12 to 2.35)	3.24 (3 to 3.49)	2.07 (1.88 to 2.27)	- 0.11 (−0.32 to 0.11)	−0.17 (−0.35 to 0.02)
Multimorbidity						
MFLE (95% CI)^3^	12.60 (12.40 to 12.79)	13.42 (13.24 to 13.59)	14.91 (14.52 to 15.30)	15.44 (15.13 to 15.73)	2.31 (2.01 to 2.62)	2.02 (1.76 to 2.29)
Proportion of life without multimorbidity	57 (56.1 to 57.9)	70.24 (69.34 to 71.14)	65.69 (59.53 to 62.69)	74.6 (73.1 to 76)	8.69 (7.40 to 9.98)	4.35 (3.02 to 5.69)
Years with multimorbidity^(1-3)^	9.5 (9.31 to 9.70)	5.68 (5.51 to 5.85)	9.49 (9.10 to 9.87)	5.26 (4.97 to 5.57)	−0.01 (−0.32 to 0.29)	−0.42 (−0.69 to −0.16)
Self-rated health						
GSR-LE (95% CI)^4^	9.18 (8.98 to 9.38)	8.78 (8.58 to 8.98)	10.99 (10.60 to 1.36)	10.32 (9.97 to 10.65)	1.82 (1.51 to 2.12)	1.54 (1.24 to 1.83)
Proportion of life free from poor health conditions	41.6 (40.7 to 42.5)	46.1 (45 to 47.1)	45 (43.41 to 46.5)	49.86 (48.16 to 51.47)	3.53 (3.23 to 3.83)	3.87 (3.57 to 4.16)
Years in poor health^(1-4)^	12.92 (12.72 to 13.12)	10.31 (10.12 to 10.52)	13.41 (13.04 to 13.80)	10.38 (10.05 to 10.73)	0.48 (0.18 to 0.79)	0.064 (−0.36 to −0.23)

*Years with disability (DLE) = Life expectancy - DFLE^(1-2)^; Years with multimorbidity (MLE) = Life expectancy - MFLE^(1-3)^; Years in poor health (PSR-LE) = Life expectancy - GSR-LE^(1-4)^.

Source: The National Household Sample Survey (2003) and the National Health Survey (2019).

As shown in Tables 4, 5, women experienced a statistically significant absolute expansion in ULE nationally and across all regions and in poor self-rated health (driven by ↑0.48 years [95% CI: 0.18 to 0.79] in poor self-rated health). In contrast, there was a statistically significant relative compression of functional disability (driven by ↑1.86% [95% CI: 0.93 to 2.79] in the proportion of years without functional disability) and multimorbidity (driven by ↑8.69% [95% CI: 7.40 to 9.98] in the proportion of years without multimorbidity).

At the national level, men showed a statistically significant relative compression of functional disability (↑1.71% [95% CI: 0.78 to 2.64] in the proportion of life without disability) and poor self-rated health (↑3.87% [95% CI: 3.57 to 4.16] in the proportion of life without poor self-rated health). There was also a statistically significant absolute compression of multimorbidity (↓0.42 years [95% CI: −0.69 to −0.16] with multimorbidity) and an absolute expansion of ULE (↑0.87 years [95% CI: 0.58 to 1.16] with ULE).

## 4 Discussion

Longevity is intrinsically linked to a population’s sociodemographic differences and health conditions. Analyzing aging in a country with such vast territorial extent as Brazil, in a way that enables an understanding of living conditions and population health, requires both national-level and regional-level investigation. Such approaches must consider the potential impacts of legislation and public health initiatives implemented during the period under review, specific populations most vulnerable to reduced longevity and illness, and the inequalities that persist in this society (26–28).

The growth in the proportion of older adults who self-identified as Black or mixed-race observed in this study does not necessarily represent an actual increase in the size of this population group. Jesus and Hoffman (29) found a 97.9% increase in the Black population between 2001 and 2015; this is speculated to be related to self-identification factors, whereby individuals stopped identifying as White and began classifying themselves as Black, driven by a sense of racial identity and belonging.

This process of identity reclassification is believed to have been influenced by the implementation of affirmative action policies, including quota systems, designed to ensure the Black population’s access to higher education and public services, as well as by legislation aimed at promoting equity and improving living conditions for Brazil’s Black population (30).

The present study found that the Brazilian older population experienced a greater increase in GSR-LE, DFLE, and MFLE than in ULE, which aligns with the findings of Zhang et al. (31). Their study on LE trends among the Chinese population between 1994 and 2015 demonstrated the positive impacts of China’s healthcare system reform, specifically improvements in the population’s cognitive and functional status.

Schmidt et al. (28) showed a reduction in HLE in the United States, which they attributed to barriers to healthcare access associated with the high cost of services, leading to greater reliance on urgent and emergency care, particularly among Black and Latino populations. In Chile, Espinoza et al. (32) observed an increase in disability and poor health among older adults despite the population living longer, identifying unequal access to the healthcare system as a potential contributing factor to this situation.

As highlighted by Cao et al. (33) in a comparative analysis of LE and HLE across 195 countries, improvements in quality of life, manifested as increased HLE, are linked to the provision of free, high-quality, and accessible health services that focus on prevention and the promotion of a healthy lifestyle.

Thus, the increase in healthy longevity observed in Brazil occurred within a context characterized by a universal, publicly funded, and free health system (SUS), as well as the development of public policies targeting older adults, including the National Health Policy for Older Adults, the Pact for Life, the International Plan of Action on Ageing, the National Health Promotion Policy, and the Statute of Older Adults, among other factors. Also noteworthy is the prioritization of longitudinal care coordinated through the Family Health Strategy, with an emphasis on access to health services and person-centered care.

In this regard, given the finding that the Brazilian population is experiencing increased healthy longevity, the study hypothesized that the reduced demand for purely curative or assistance-based care for older adults is linked to the development of health actions and policies centered on primary care. Strengthening health promotion and disease prevention through the reallocation of public health spending and increased financial investment in these areas broadens the scope of healthcare beyond urgent and emergency situations (4) by consolidating longitudinal care.

On the other hand, the relative compression of life expectancy with disability (DLE), coupled with an absolute increase in poor self-rated health, suggests that, although older Brazilian individuals experience fewer years with disability, their health perception does not align with this reduction in disability. The absolute rise in multimorbidity confirms Brazil’s status as a country undergoing an epidemiological transition, characterized by the emergence of chronic degenerative diseases and non-fatal health conditions.

This finding is consistent with the “healthy survivor effect” observed by Head et al. (34) in a study conducted in England; this concept is based on the understanding that individuals in better health tend to live longer. Consequently, the observed increase in the prevalence of multimorbidity among the English population was associated with the earlier onset of multimorbidity and a reduction in its lethality.

This study identified disparities between the North and Northeast and South and Southeast regions of Brazil, reflecting the historical concentration of health services in the South and Southeast regions (27, 35). Populations in these regions enjoy greater access to diagnostic services and a higher ratio of physicians per 1,000 inhabitants (35, 36). As observed in this study, this fosters a reduction in functional disability and promotes better self-rated health among residents compared to other regions.

It is crucial to emphasize that a country’s socioeconomic inequities drive disparities in healthcare access and, in turn, contribute to a higher prevalence of poor self-rated health among the economically vulnerable population (37). However, a comparison between 2003 and 2019 revealed that the North region experienced the greatest increase in MFLE, demonstrating that population aging in this region is occurring rapidly and is accompanied by improvements in health. Studies by Neves et al. (38) covering 2006–2016 and Giovanella et al. (39) covering 2013–2019 showed that healthy aging in the North region has progressed at a pace similar to the expansion of Family Health Strategy coverage. Hence, further research is needed for a more in-depth analysis of the potential relationship between the rise in MFLE and the expansion of Family Health Strategy coverage.

Furthermore, the increase in MFLE warrants closer examination, as a lower probability of chronic disease diagnosis may be partly linked to limited access to health services, potentially reflecting regional differences in diagnostic detection (35, 36).

Public health spending has the potential to reduce health and regional inequalities. Thus, it is essential to adapt policies to the reality of each region and ensure an equitable distribution of public spending, thereby strengthening public health (40, 41).

This finding is consistent with the study by Corris et al. (42), who found that the unequal distribution of health services and spending negatively affected HLE among the English population. Similarly, in a study involving the African population, Koroglu and Bayar (41) found that increased health spending was associated with improvements in HLE indicators; however, they also emphasized the need for health policies that promote equitable access to healthcare.

In light of the demographic transition occurring in Brazil and worldwide, characterized by a growing older population, the study by Xi et al. (43) on the global burden of disease demonstrated the essential need for future policies to address these changes by aligning health investment with the population’s needs.

Austerity policies based exclusively on controlling social security spending, such as those implemented in the United Kingdom, neglect the most vulnerable populations and exacerbate inequalities in the distribution of health services and spending, thereby undermining healthy, well-being-oriented population aging (42, 44).

In this context, Welsh et al. (45) highlighted that the United Kingdom has redirected its policies following this experience to reduce health inequalities and increase HLE by 2035 through investments and the development of targeted health policies.

Population aging in Brazil is characterized by gender-based disparities: Women have longer LE than men, yet these additional years are not spent in good health, a finding consistent with the results of Szwarcwald et al. (46). Although men have shorter LE, they spend a greater proportion of their lives in good health, illustrating the gender-based health survival paradox, where women live longer but spend more years in poor health. Lu et al. (47) corroborated the sex differences identified in the Brazilian population, finding that older men in China have longer DFLE; thus, despite living longer, women experience a poorer quality of life.

Zhang et al. (31), Solé-Auró et al. (48), and Nielsen et al. (49) found that women are becoming less healthy at older ages, possibly due to the accumulation of family and professional responsibilities, which leads to overload and progressive illness.

Zarulli et al. (50) analyzed population survival under extreme conditions (pandemics, famine, and slavery) and found that factors contributing to higher male mortality are linked to riskier behaviors (e.g., greater alcohol and psychoactive substance consumption) compared to women. The authors also highlighted the role of genetic factors, explaining the potential link between certain pathologies and testosterone, which characterizes differential mortality selection. Men report fewer illnesses and limitations at older ages (reporting bias) and possess greater physical strength than women (51), reinforcing the health survival paradox.

The absolute compression of MLE and the relative compression of PSR-LE and DLE among older Brazilian individuals demonstrate that, following survival beyond the primary causes of male mortality in youth (including external causes such as assault, violence, and traffic accidents), male mortality rates decline. Consequently, men are experiencing increased healthy longevity and tend to live in better health (52).

Thus, given that the Brazilian population is living longer and healthier lives, extending HLE is directly linked to analyzing the aforementioned gender disparities to ensure better living conditions and population health. To this end, a critical analysis of existing health policies is essential, supported by studies that delve into this topic from this perspective and drive further progress toward ensuring healthy longevity.

Based on the findings, it is necessary to outline the study’s potential limitations. These include the exclusion of the institutionalized Brazilian population from the survey sample, which directly affects the characteristics of the study population, given the greater frailty of this group; the omission of diseases affecting older adults (e.g., Alzheimer’s disease and Parkinson’s disease), whose rising prevalence has established them as public health issues; survivorship bias, given that the data are cross-sectional (although most studies on HLE adopt a similar approach) (14, 31, 49); the large number of comparisons across sex, region, and year without adjustments for multiple comparisons (common in descriptive studies on HLE), which increases the likelihood of Type I error, meaning some statistically significant differences may have occurred by chance; and the use of a proxy informant, whose data provided during the surveys may present inconsistencies and discrepancies for analysis.

This study stands out due to several key strengths. It was conducted using a population that is representative at both the national and regional levels, with surveys carried out by national agencies. It provides the most recent data on HLE, DFLE, MFLE, and GSR-LE for a country that accounts for nearly half of South America’s land area and population, making Brazil a significant case study. It identifies disparities in the aging process by sex and examines trends in HLE in Brazil, paralleling patterns seen in developed nations, which can inform the restructuring of Brazilian public policies by drawing lessons from the successes and setbacks experienced by countries that faced population aging earlier. Finally, the study employed an innovative approach to MFLE by adjusting morbidity incidence to the physiological changes associated with aging. Hence, the study identifies the impact of co-existing conditions on the health of this population group, thereby enabling a more accurate analysis of HLE among older adults.

Ensuring improvements in the Brazilian population’s quality of life and healthy and sustainable aging requires public health and its associated legal frameworks to serve as guiding principles. HLE findings from this study demonstrated that older Brazilian individuals experienced an increase in HLE during the analyzed period, characterized by an absolute expansion in DFLE, GSR-LE, and MFLE. However, although women exhibited higher overall LE and HLE than men, they also showed a statistically significant increase in ULE that was greater than that observed in men.

Consequently, the proper planning, formulation, and implementation of comprehensive Brazilian socioeconomic policies and strategies aimed at preventing illness, providing treatment, and promoting the population’s general well-being (52) must analyze and consider the gender-based health survival paradox and the need for equitable public spending. Such an approach is expected to lead to appropriate public spending allocation and a reduction in inequalities, thereby providing dignified living conditions and healthcare for the older Brazilian population and, ultimately, improving the quality of life of this demographic group.

## Data Availability

The datasets presented in this study can be found in online repositories. The names of the repository/repositories and accession number(s) can be found at: www.ibge.gov.br/estatisticas/sociais/populacao/19898-suplementos-pnad3.html?edicao=10409&t=microdados and www.ibge.gov.br/estatisticas/sociais/saude/9160-pesquisa-nacional-de-saude.html?=&t=downloads.
