# Supplementary material for: Healthy life expectancy and its trends among Brazilian older adults from 2003 to 2019: an observational study using PNAD and PNS data
Source: Front Public Health. 2026 Oct 19;14:1797715. doi: 10.3389/fpubh.2026.1797715 (PMC13724616; doi:10.3389/fpubh.2026.1797715)
Supplement: Supplementary file 1 [file Table_1.DOC]

***Supplementary Material***

1 **Supplementary Tables**

**Supplementary Table 1. Life expectancy and years lived in unhealthy states by age, Brazil, 2003 and 2019.**

| **AGE** | **LE 2003** | **LE 2019** | **PSR-LE 2003** | **PSR-LE 2019** | **DLE 2003** | **DLE 2019** | **MLE 2003** | **MLE 2019** | **ULE 2003** | **ULE 2019** |
| --- | --- | --- | --- | --- | --- | --- | --- | --- | --- | --- |
| 60 | 20.60 | 22.70 | 2.78 | 2.55 | 2.73 | 2.76 | 7.93 | 7.61 | 9.36 | 9.99 |
| 65 | 17.20 | 18.90 | 2.56 | 2.23 | 2.72 | 2.80 | 7.03 | 6.85 | 8.52 | 8.95 |
| 70 | 14.10 | 15.50 | 2.36 | 1.98 | 2.74 | 2.85 | 6.00 | 5.90 | 7.53 | 7.8 |
| 75 | 11.50 | 12.40 | 2.24 | 1.7 | 2.82 | 3.01 | 4.86 | 4.96 | 4.95 | 6.71 |
| 80+ | 9.30 | 9.70 | 2 | 1.33 | 2.81 | 3.12 | 3.6 | 4 | 3.63 | 5.49 |

* Life expectancy (LE);

Years lived in poor self-rated health (PSR-LE) = Life expectancy - GSR-LE;

Years lived with functional disability (DLE) = Life expectancy - DFLE;

Years lived with multimorbidities (MLE)= Life expectancy - MFLE;

Unhealthy life expectancy (ULE) = Life expectancy – HLE.

**Supplementary Table 2. Healthy life expectancy (HLE) at age 60 according to self-rated health, functional disability, and multimorbidity, by sex and geographic region, Brazil, 2003–2019.**

| **2003** | |  | **2008** | | **2013** |  | **2019** |  | **Difference between 2019-2003** | |
| --- | --- | --- | --- | --- | --- | --- | --- | --- | --- | --- |
|  | **Woman** | **Man** | **Woman** | **Man** | **Woman** | **Man** | **Woman** | **Man** | **Woman** | **Man** |
| **BRAZIL** |  |  |  |  |  |  |  |  |  |  |
| Life expectancy (years) ^1^ | 22.1 | 19.1 | 22.7 | 19.5 | 23.5 | 19.9 | 24.4 | 20.7 | 2.3 | 1.6 |
| HLE (95%CI) ^2^ | 6.66  (6.49 to 6.86) | 7.43  (7.24 to 7 .62) | 7.10  (6.95 to 7.26) | 7,37  (7.22 to 7.55) | 7.28  (6.85 to 7.70) | 7.46  (6.98 to 7.94) | 7.43  (7.0 8 to 7.77) | 8.17  (7.83 to 8.48) | 0.76  (0.48 to 1.05) | 0.73  (0.44 to 1.02) |
| Healthy life proportion | 30.2  (29.4 to 31.1) | 38.91  (37.92 to 39.91) | 31.3  (30.6 to 32) | 37.8  (37 to 38.7) | 31  (29.2 to 32.8) | 37.5  (35.1 to 39.9) | 30.44  (29.04 to 31.84) | 39.45  (37.85 to 40.95) | 0.29  (0.01 to 0.57) | 0.54  (0.25 to 0.83) |
| Unhealthy years ^(1-2)^ | 15.44  (15.24 to 15.61) | 11.67  (11.48 to 11.86) | 15.59  (15.43 to 15,75) | 12,10  (11.93 to 12.26) | 16.20  (15.77 to 16.62) | 12.44  (11.96 to 12.92) | 16.97  (16.63 to 17.31) | 12.53  (12.22 to 12.86) | 1.53  (1.25 to1.82) | 0.87  (0.58 to 1.16) |
| **NORTH** |  |  |  |  |  |  |  |  |  |  |
| Life expectancy (years) 3 | 19.64 | 17.19 | 20.74 | 18.11 | 21.6 | 18.72 | 22.41 | 19.17 | 2.77 | 1.98 |
| HLE (95%CI) ^4^ | 5.07  (4.52 to 5.64) | 5.26  (4.61 to 5.96) | 6.06  (5.52 to 6.61) | 5.56  (5.11 to 6.03) | 5.85  (4.80 to 7.06) | 6.05  (4.83 to 7.41) | 6.61  (5.85 to 7.40) | 7.03  (6.32 to 7.78) | 1.54  (0.77 to 2.32) | 1.77  (1.03 to 2.52) |
| Healthy life proportion | 25.81  (23.01 to 28.71) | 30.60  (26.8 to 34.7) | 29..2  (26.6to 31.9) | 30.7  (28.2 to 33.3) | 27.1  (22.2 to 32.7) | 32.3  (25.8 to 39.6) | 29.51  (26.11 to 33.01) | 36.70  (33 to 40.60) | 3.70  (2.93 to 4.47) | 6.09  (5.33 to 6.84) |
| Unhealthy years ^(3-4)^ | 14.57  (14 to 15.12) | 11.93  (11.22 to 12.58) | 14.68  (14.12 to 15.22) | 12.55  (12.08 to 13) | 15.75  (14.54 to 16.81) | 12.67  (11.31 to 13.89) | 15.80  (15.01 to 16.56) | 12.13  (11.39 to 12.84) | 1.23  (0.45 to 2) | 0.21  (-0.54 to 0.95) |
| **NORTH EAST** |  |  |  |  |  |  |  |  |  |  |
| Life expectancy (years) ^5^ | 20.68 | 17.54 | 21.53 | 18.22 | 22.36 | 18.86 | 23.26 | 19.45 | 2.58 | 1.91 |
| HLE (95%CI) ^6^ | 5.15  (4.82 to 5.52) | 5.87  (5.51 to 6.24) | 5.79  (5.56 to 6.03) | 5.79  (5.54 to 6.07) | 5.16  (4.58 to 5.79) | 6.32  (5.58 to 7.11) | 5.56  (5.07 to 6.09) | 6.28  (5.82 to 6.75) | 0.41  ( -0.02 to 0.84) | 0.41  (-0.04 to 0.86) |
| Healthy life proportion | 24.90  (23.30 to 26.70) | 33.49  (31.39 to 35.59) | 26.9  (25.8 to 28) | 31.8  (30.4 to 33.3) | 23.1  (20.5 to 25.9) | 33.5  (29.6 to 37.7) | 23.90  (21.8 to 26.2) | 32.30  (29.9 to 34.7) | -0.99  (-1.43 to -0.56) | -1.19  (-1.65 to -0.74) |
| Unhealthy years ^(5-6)^ | 15.53  (15.16 to 15.86) | 11.66  (11.30 to 12.03) | 15.74  (15.50 to 15.98) | 12.42  (12.15 to 12.68) | 17.19  (16.57 to 17.78) | 12.54  (13.28 to 11.75) | 17.70  (17.16 to 18.19) | 13.17  12.7 to 13.63) | 2.17  (1.74 to 2.60) | 1.50  (1.05 to 1.95) |

| **2003** | |  | **2008** | | **2013** |  | **2019** | | **Difference between 2019-2003** | |
| --- | --- | --- | --- | --- | --- | --- | --- | --- | --- | --- |
|  | **Woman** | **Man** | **Woman** | **Man** | **Woman** | **Man** | **Woman** | **Man** | **Woman** | **Man** |
| **SOUTHEAST** |  |  |  |  |  |  |  |  |  |  |
| Life expectancy (years) ^7^ | 22.18 | 18.32 | 23.16 | 19.44 | 24.06 | 20.43 | 24.98 | 21.39 | 2.8 | 3.07 |
| HLE (95%CI) ^8^ | 7.54  (7.27 to 7.83) | 8.02  (7.77 to 8.30) | 8.06  (7.81 to 8.34) | 8.14  (7.87 to 8.42) | 8.61  (7.91 to 9.36) | 8.23  (7.40 to 9.09) | 8.27  (7.67 to 8.89) | 9.26  (8.66 to 9.86) | 0.73  (0.19 to 1.27) | 1.24  (0.68 to 1.80) |
| Healthy life proportion | 33.99  (32.79 to 35.29) | 43.79  (42.40 to 45.29) | 34.8  (33.7 to 36) | 41.9  (40.5 to 43.3) | 35.8  (32.9 to 38.9) | 40.30  (36.2 to 44.56) | 33.10  (30.70 to 35.6) | 43.3  (40.51 to 46.11) | -0.89  (-1.43 to -0.35) | -0.49  (-0.88 to -0.18) |
| Unhealthy years ^(7-8)^ | 14.64  (14.35 to 14.91) | 10.30  (10.02 to 10.55) | 15.10  (14.82 to 15.36) | 11.29  (11.02 to 11.57) | 15.44  (14.70 to 16.14) | 12.20  (11.34 to 13.03) | 16.71  (16.09 to 17.31) | 12.13  (11.53 to 12.73) | 2.07  (1.53 to 2.61) | 1.83  (1.27 to 2.39) |
| **SOUTH** |  |  |  |  |  |  |  |  |  |  |
| Life expectancy (years) ^9^ | 21.71 | 18.03 | 23.03 | 19.38 | 24.17 | 20.39 | 25.33 | 21.29 | 3.62 | 3.26 |
| HLE (95%CI) ^10^ | 6.40  (5.93 to 6.88) | 6.83  (6.44 to 7.27) | 6.82  (6.45 to 7.23) | 7.62  (7.19 to 8.04) | 7.25  (6.24 to 8.36) | 7.24  (6.22 to 8.34) | 8.41  (7.57 to 9.27) | 8.66  (7.96 to 9.37) | 2  (1.27 to 2.74) | 1.81  (1.09 to 2.54) |
| Healthy life proportion | 29.50  (27.30 to 31.70) | 38.01  (35.71 to 40.31) | 29.6  (28 to 31.4) | 39.3  (37.1 to 41.5) | 30  (25.82 to 34.6) | 35.5  (30.5 to 40.9) | 33.19  (29.89 to 36.59) | 40.70  (37.14 to 44) | 3.70  (2.97 to 4.43) | 2.69  (1.96 to 3.42) |
| Unhealthy years ^(9-10)^ | 15.31  (14.83 to 15.78) | 11.18  (10.76 to 11.59) | 16.21  (15.80 to 16.58) | 11.76  (11.34 to 12.19) | 16.92  (15.81 to 17.94) | 13.15  (12,.05 to 14.17) | 16.93  (16.06 to 17.76) | 12.63  (11.92 to 13.33) | 1.62  (0.88 to 2.35) | 1.45  (0.72 to 2.17) |
| **CENTRAL to WEST** |  |  |  |  |  |  |  |  |  |  |
| Life expectancy (years) ^11^ | 21.2 | 18.5 | 22.07 | 19.25 | 22.85 | 19.83 | 23.68 | 20.39 | 2.48 | 1.89 |
| HLE (95%CI) ^12^ | 5.87  (5.43 to 6.32) | 6.59  (6.05 to 7.16) | 6.13  (5.67 to 6.60) | 6.89  (6.47 to 7.33) | 6.99  (6.08 to 7.97) | 8.25  (7.18 to 9.38) | 7.06  (6.18 to 7.98) | 7.99  (7.19 to 8.81) | 1.18  (0.30 to 2.07) | 1.41  (0.52 to 2.29) |
| Healthy life proportion | 27.70  (25.60 to 29.80) | 35.60  (32.7 to 38.7) | 27.8  (25.7 to 29.9) | 35.8  (33.6 to 38.1) | 30.6  (26.6 to 34.9) | 41.6  (36.2 to 47.3) | 29.80  (26.10 to 33.7) | 39.20  (35.3 to 43.2) | 2.10  (1.21 to 2.98) | 3.60  (2.70 to 4.50) |
| Unhealthy years ^(11-^ ^12)^ | 15.33  (14.88 to 15.77) | 11.91  (11.34 to 12.45) | 15.93  (15.47 to 16.40) | 11.87  (11.45 to 12.28) | 15.85  (14.87 to 16.77) | 11.58  (10.45 to 12.65) | 16.62  (15.70 to 17.5) | 12.40  (11.58 to 13.19) | 1.30  (0.41 to 2.18) | 0.48  (-0.40 to .37) |

* Unhealthy years = Life expectancy - HLE

Source: National Household Sample Survey (2003 and 2008). National Health Survey (2013 and 2019).

**Supplementary Table 3. Disability-free life expectancy (DFLE), multimorbidity-free life expectancy (MFLE), and life expectancy with good self-rated health (GSR-LE) at age 60, by sex and geographic region, Brazil, 2003–2019.**

| **2003** | |  | **2008** | | **2013** |  | **2019** | | **Difference between 2019-2003** | |
| --- | --- | --- | --- | --- | --- | --- | --- | --- | --- | --- |
|  | **Woman** | **Man** | **Woman** | **Man** | **Woman** | **Man** | **Woman** | **Man** | **Woman** | **Man** |
| **FUNCTIONAL DISABILITY** | | | | | | | | | | |
| Life expectancy (years) ^1^ | 22.1 | 19.1 | 22.7 | 19.5 | 23.5 | 19.9 | 24.4 | 20.7 | 2.3 | 1.6 |
| DFLE (95%CI) ^2^ | 18.75  (18.60 to 18.90) | 16.87  (16.75 to 16.98) | 18.84  (18.68 to 18.97) | 16.97  (16.85 to 17.07) | 21.03  (20.75 to 21.31) | 18.29  (18.01 to 18.55) | 21.16  (20.91 to 21.40) | 18.63  (18.43 to 18.82) | 2.40  (2.19 to 2.62) | 1.77  (1.58 to 1.95) |
| Proportion of life without disability | 84.85  (84.15 to 85.54) | 88.30  (87.7 to 88.9) | 82.99  (82.29 to 83.59) | 87.03  (86.43 to 87.53) | 89.49  (88.29 to 90.69) | 91.93  (90.53 to 93.23) | 86.71  (85.7 to 87.7) | 90.02  (89.01 to 90.92) | 1.86  (0.93 to 2.79) | 1.71  (0.78 to 2.64) |
| Years with disability  (1-2) | 3.35  (3.19 to 3.50) | 2.23  (2.12 to 2.35) | 3.86  (3.72 to 4.02) | 2.53  (2.43 to 2.65) | 2.47  (2.19 to 2.75) | 1.61  (1.35 to 1.89) | 3.24  (3 to 3.49) | 2.07  (1.88 to 2.27) | - 0.11  (- 0.32 to 0.11) | - 0.17  (-0.35 to 0.02) |
| **MULTIMORBIDITY** | | | | | | | | | | |
| MFLE (95%CI)^3^ | 12.60  (12.40 to 12.79) | 13.42  (13.24 to 13.59) | 12.85  (12.66 to 13.03) | 13.54  (13.39 to 1368) | 15.11  (14.61 to 15.61) | 14.35  (13.83 to 14.83) | 14.91  (14.52 to 15.30) | 15.44  (15.13 to 15.73) | 2.31  (2.01 to 2.62) | 2.02  (1.76 to 2.29) |
| Proportion of life without  multimorbidities | 57  (56.1 to 57.9) | 70.24  (69.34 to 71.14) | 56.59  (55.79 to 57.39) | 69.46  (68.66 to 70.16) | 69.31  (62.17 to 66.42) | 72.12  (69.52 to 74.52) | 65.69  (59.53 to 62.69) | 74.6  (73.1 to 76) | 8.69  (7.40 to 9.98) | 4.35  (3.02 to 5.69) |
| Years with multimorbidities ^(1-3)^ | 9.5  (9.31 to 9.70) | 5.68  (5.51 to 5.85) | 9.85  (9.67 to 10.03) | 5.96  (5.82 to 6.11) | 8.39  (7.89 to 8.89) | 5.55  (5.07 to 6.07) | 9.49  (9.10 to 9.87) | 5.26  (4.97 to 5.57) | -0.01  (- 0.32 to 0.29) | - 0.42  (- 0.69 to -0.16) |
| **SELF-RATED HEALTH** | | | | | | | | | | |
| GSR-LE  (95%CI) ^4^ | 9.18  (8.98 to 9.38) | 8.78  (8.58 to 8.98) | 10.15  (9.96 to 10.33 ) | 8.87  (8.70 to 9.03) | 9.98  (9.51 to 10.42) | 9.33  (8.84 to 9.81) | 10.99  (10.60 to 1.36) | 10.32  (9.97 to 10.65) | 1.82  (1.51 to 2.12) | 1.54  (1.24 to 1.83) |
| Proportion of life free from poor health conditions | 41.6  (40.7 to 42.5) | 46.1  (45 to 47.1) | 44.7  (43.9 to 45.5) | 45.5  (44.6 to 46.3) | 42.5  (40.5 to 44.4) | 46.92  (44.4 to 49.3) | 45  (43.41 to 46.5) | 49.86  (48.16 to 51.47) | 3.53  (3.23 to 3.83) | 3.87  (3.57 to 4.16) |
| Years in poor health  (1-4) | 12.92  (12.72 to 13.12) | 10.31  (10.12 to 10.52) | 12.55  (12.37 to 12.73) | 10.61  (10.45 to 10.78) | 13.50  (13.05 to 13.97) | 10.33  (9.87 to 10.82) | 13.41  (13.04 to 13.80) | 10.38  (10.05 to 10.73) | 0.48  (0.18 to 0.79) | 0.064  (-0.36 to -0.23) |

*Years with disability (DLE) = Life expectancy - DFLE ^(1-2)^; Years with multimorbidities (MLE)= Life expectancy - MFLE ^(1-3)^ ; Years in poor self-rated health (PSR-LE)= life expectancy - GSR-LE ^(1-4).^

Source: National Household Sample Survey (2003 and 2008). National Health Survey (2013 and 2019).

1 **Supplementary Figure 1**

| **Variable** | **PNAD 2003** | **PNAD 2008** | **PNS 2013** | **PNS 2019** |
| --- | --- | --- | --- | --- |
| **Self-rated health** | V1303- In general, consider their own state of health as | v1303- In general, consider their own state of health as | N001 - In general, how do you rate your health? | N001 - In general, how do you rate your health? |
| **Functional disability** | V1403- Usually, due to health problems, has difficulty eating, bathing or going to the toilet. | V1403 - Usually, due to health problems, has difficulty eating, bathing or going to the toilet. | K001 - In general, how difficult is  to eat alone with a plate in front of you, including holding a fork, cutting food and drinking from a glass? | K001 - In general, how difficult is to eat alone with a plate in front of you, including holding a fork, cutting food and drinking from a glass? |
|  |  |  | K004 - In general, how difficult is it for  to bathe alone, including getting in and out of the shower or bath | K004 - In general, how difficult is it for to bathe alone, including getting in and out of the shower or bath? |
|  |  |  | K007 - In general, how difficult is  to go to the toilet by yourself, including sitting down and getting up? | K007 - In general, how difficult is to go to the toilet by yourself, including sitting down and getting up? |

| **Chronic diseases** | Some doctor or health professional has already said that... you do:  v1310 - Arthritis or rheumatism  v1311 - Cancer? v1312 - Diabetes?  v1313 - Bronchitis or asthma?  v1314 - Hypertension (high blood pressure)?  v1315- Heart disease?  v1316 - Chronic renal failure? v1317 - Depression? | Some doctor or health professional has already said that... you do:  v1310 - Arthritis or rheumatism  v1311 - Cancer?  v1312 - Diabetes?  v1313 - Bronchitis or asthma?  v1314 - Hypertension (high blood pressure)?  v1315- Heart disease?  v1316 - Chronic renal failure?  v1317 - Depression? | Have you ever been diagnosed by a doctor?  Q002 - Hypertension (high blood pressure)?  Q030a - Diabetes? Q063a - Heart attack? Q063a - Heart failure?  Q74 - Asthma (or asthmatic bronchitis)?  Q79 - Arthritis or rheumatism? Q92 - Depression?  Q116a - Pulmonary emphysema? Q120 - Cancer?  Q124 - Chronic renal failure? | Have you ever been diagnosed by a doctor?:  Q002 - Hypertension (high blood pressure)?  Q030a - Diabetes? Q063a - Heart attack? Q063a - Heart failure?  Q74 - Asthma (or asthmatic bronchitis)?  Q79 - Arthritis or rheumatism?  Q92 - Depression?  Q116a - Pulmonary emphysema?  Q120 - Cancer?  Q124 - Chronic renal failure? |
| --- | --- | --- | --- | --- |

**Supplementary Figure 1. Survey questions from PNAD 2003 and 2008 and PNS 2013 and 2019 used to assess health status among the Brazilian population aged 60 years and older.**

Source: National Sample Survey (2003 and 2008). National Health Survey (2013 and 2019).

Note: During the analysis of the 2008 and 2013 data, particular attention was paid to the transition from a broader survey framework to a survey focused exclusively on health, in order to ensure comparability and harmonization across survey waves. The following procedures were therefore adopted:

- Functional disability: In 2003 and 2008, functional disability was assessed using a single question (v1403), whereas in 2013 it was assessed through three questions (K001, K004, and K007). To harmonize this variable across survey waves, a composite variable was constructed based on the three questions from the 2013 PNS, following the functional classification described in the methodological notes of the 2008 PNAD. Individuals who reported any degree of difficulty performing at least one of the three assessed activities were classified as having functional disability.
- Chronic diseases: In both surveys, according to the methodological notes of the 2008 PNAD and the PNS 2013 questionnaire manual, chronic disease status was defined based on the presence of a reported diagnosis.

2 **Supplementary Figure 2**

| **Dimension of Analysis** | **Wording in the PNAD**  **(2003 and 2019)** | **Wording in the PNS**  **(2013 and 2019)** | **Harmonization Decision** | **Probable Direction of the Residual Bias** |
| --- | --- | --- | --- | --- |
| **Self-Rated Health** | V1303- In general, consider their own state of health as | N001 - In general, how do you rate your health? | Creation of a binary variable:  Good self-rated health (Very good/Good) vs.  Poor self-rated health (Fair/Poor/Very poor). | **Potential overestimation of good self-rated health in 2003–2008:** More favorable ratings by proxy respondents may have led to an overestimation of good self-rated health. |
| **Multimorbidity** | Some doctor or health professional has already said that... you do:  v1310 - Arthritis or rheumatism v1311 - Cancer?  v1312 - Diabetes?  v1313 - Bronchitis or asthma? v1314 - Hypertension (high blood pressure)?  v1315- Heart disease?  v1316 - Chronic renal failure? v1317 - Depression? | Have you ever been diagnosed by a doctor?  Q002 - Hypertension (high blood pressure)?  Q030a - Diabetes? Q063a - Heart attack? Q063a - Heart failure?  Q74 - Asthma (or asthmatic bronchitis)?  Q79 - Arthritis or rheumatism? Q92 - Depression?  Q116a - Pulmonary emphysema?  Q120 - Cancer?  Q124 - Chronic renal failure? | Creation of a binary variable:   - Without multimorbidity (with 1 disease) - With multimorbidity (with 2 or more diseases) using only common conditions | **Potential underestimation of multimorbidity in 2003–2008:** Limited knowledge of diagnoses among proxy respondents may have resulted in underreporting and lower estimated multimorbidity prevalence. |
| **Functional disability** | V1403- Usually, due to health problems, has difficulty eating, bathing or going to the toilet. | K001 - In general, how difficult is to eat alone with a plate in front of you, including holding a fork, cutting food and drinking from a glass?  K004 - In general, how difficult is it for to bathe alone, including getting in and out of the shower or bath  K007 - In general, how difficult is to go to the toilet by  yourself, including sitting down and getting up? | Compare only identical items related to mobility and personal care (e.g., bathing, eating, walking), categorizing them as follows:   - No disability (no difficulty) - With disability (unable to do so/has great difficulty/has slight difficulty). | **Potential underestimation of functional disability in 2003–2008:** Underreporting of moderate difficulties by proxy respondents may have led to an underestimation of functional disability. |

**Supplementary Figure 2. Survey questions from PNAD 2003 and 2008 and PNS 2013 and 2019 used to assess health status among the Brazilian population aged 60 years and older, including harmonization strategies and potential sources of residual bias in the assessment of functional disability, multimorbidity, and self-rated health.**
